# Metabolic pathway for degradation of 2-chloro-4-aminophenol by *Arthrobacter* sp. SPG

**DOI:** 10.1186/s12934-014-0164-6

**Published:** 2014-11-27

**Authors:** Pankaj Kumar Arora, Tapan Kumar Mohanta, Alok Srivastava, Hanhong Bae, Vijay Pal Singh

**Affiliations:** School of Biotechnology, Yeungnam University, Gyeongsan, 712-749 Republic of Korea; Department of Plant Science, Faculty of Applied Sciences, MJP Rohilkhand University, Bareilly, 243006 India

## Abstract

A degradation pathway of 2-chloro-4-aminophenol (2C4AP) was studied in an *Arthrobacter* sp. SPG that utilized 2C4AP as its sole source of carbon and energy. The 2C4AP degradation was initiated by a 2C4AP-deaminase that catalyzed the conversion of 2C4AP into chlorohydroquinone (CHQ) with removal of ammonium ion. In the next step, a CHQ-dehalogenase dehalogenated CHQ to hydroquinone (HQ) that cleaved into γ-hydroxymuconic semialdehyde by a HQ-dioxygenase. The 2C4AP degradation was also investigated in sterile and non-sterile soil microcosms using strain SPG. The results show that the SPG cells degraded 2C4AP more rapidly in sterile soil than non-sterile soil. Our studies showed that strain SPG may be used for bioremediation of 2C4AP-contaminated sites. This is the first report of the 2C4AP degradation by any bacteria.

## Introduction

Chloroaminophenols (CAPs) including 2-chloro-4-aminophenol (2C4AP) and 4-chloro-2-aminophenol (4C2AP) are widely used as hair dyes and have been classified as toxic substances because of their carcinogenicity [1]. CAPs have been released into soil and water as the by-products during the synthesis of cosmetic dyes and chemicals. They may also release into soil because of microbial degradation of various chemicals including 3-chloronitrobenzene and 4-chloro-2-nitrophenol (4C2NP) [2,3].

Few bacteria capable of utilizing CAPs as their sole sources of carbon and energy have been identified and characterized [1,4]. Examples are *Exiguobacterium* sp. PMA [4] and *Burkholderia* sp. RKJ 800 [1]. Both of the bacterial strains utilized 4C2AP as the sole source of carbon and energy. The complete mineralization of 4C2AP was studied in *Burkholderia* sp. RKJ 800 that degraded it with release of ammonium and chloride ions [1]. The 4C2AP degradation was initiated with the formation of 4-chlorocatechol that was further degraded via ring cleavage [1].

The bacterial degradation of CAPs may be initiated by one of the following mechanisms: (i) the removal of ammonium ion from a CAP by a deaminase [1]; (ii) the ring cleavage of a CAP by an aminophenol dioxygenase [5]; (ii) the dehalogenation of a CAP by a dehalogenase [4]; and (iii) the acetylation of a CAP [2,3,6].

In this communication, we have reported degradation of 2C4AP by *Arthrobacter* sp. SPG. Strain SPG was previously isolated from the soil collected from a pesticide contaminated site, India by an enrichment method using 4-nitrophenol [7]. Strain SPG utilized 4-nitrophenol, 2-chloro-4-nitrophenol (2C4NP), 2-nitrobenzoate, 3-methyl-4-nitrophenol and nitrocatechol as its sole sources of carbon and energy [7]. In addition, *Arthrobacter* sp. SPG is also capable of utilizing 2C4AP as its sole source of carbon and energy [8]. The aim of this work is to study the degradation pathway of 2C4AP by *Arthrobacter* sp. strain SPG and monitor the 2C4AP degradation potential of the same strain in soil under laboratory conditions.

## Results

### Growth and degradation studies

**Figure 1:**
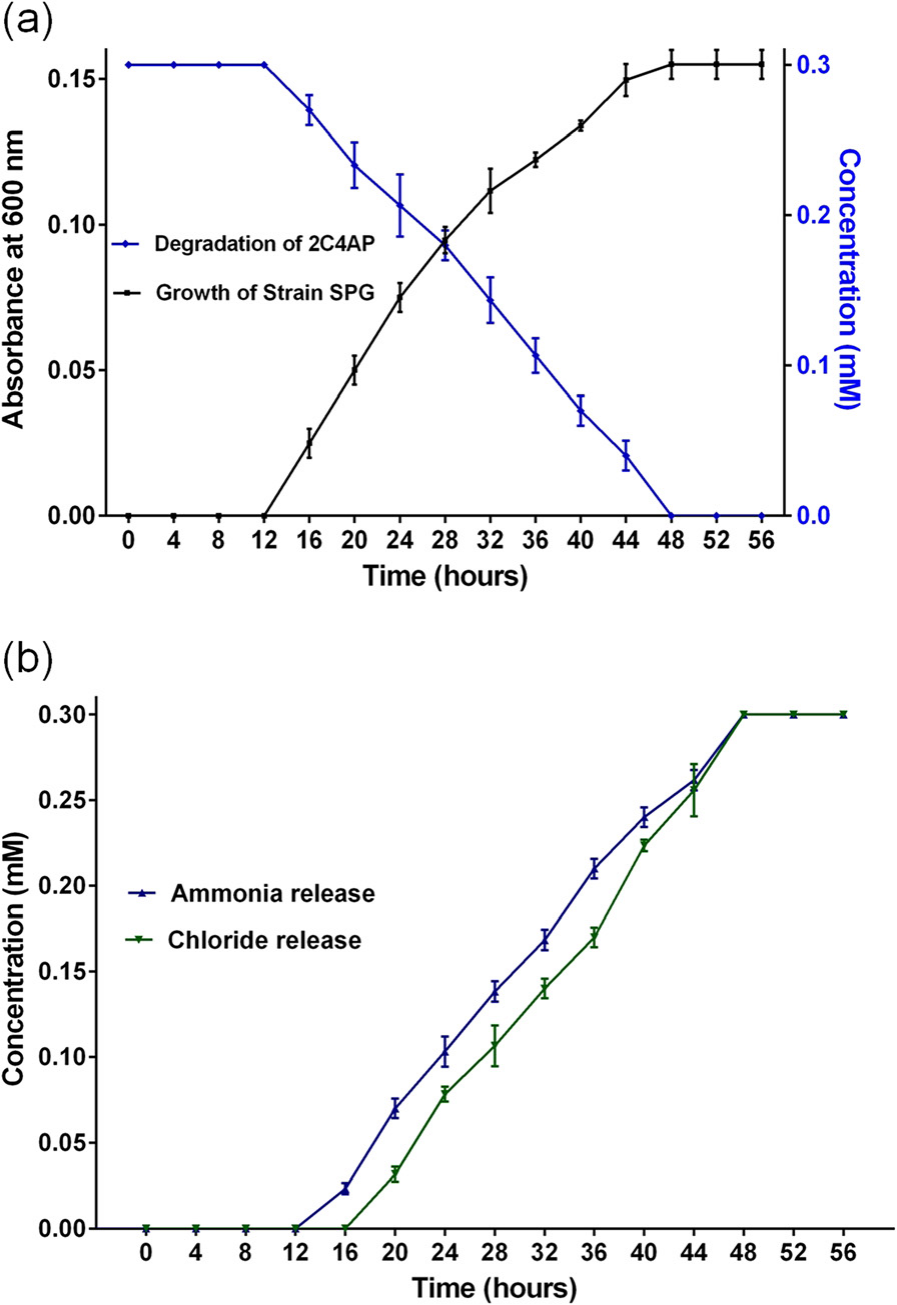
**Growth and degradation studies. (a)** Utilization and degradation of 2-chloro-4-aminophenol by *Arthrobacter* sp. SPG, and **(b)** Estimation of ammonium and chloride ions during the degradation of 2-chloro-4-aminophenol by *Arthrobacter* sp. SPG.

*Arthrobacter* sp. SPG uses 0.3 mM 2C4AP as its sole source of carbon and energy and mineralized 2C4AP completely within 48 h (Figure 1a). The growth of the cells of strain SPG initiated after the incubation of 12 h and the maximum absorbance at 600 nm reached was 0.15 within 48 hours of incubation. The cells of strain SPG released stoichiometric amounts of chloride and ammonium ions during the degradation of 2C4AP. The time course analysis of ammonia and chloride releases showed that the initiation of the ammonia release occurred before the initiation of the chloride release. These data suggest that the 2C4AP degradation was initiated with ammonia release and followed by chloride release (Figure 1b).

### Identification of metabolites

**Figure 2:**
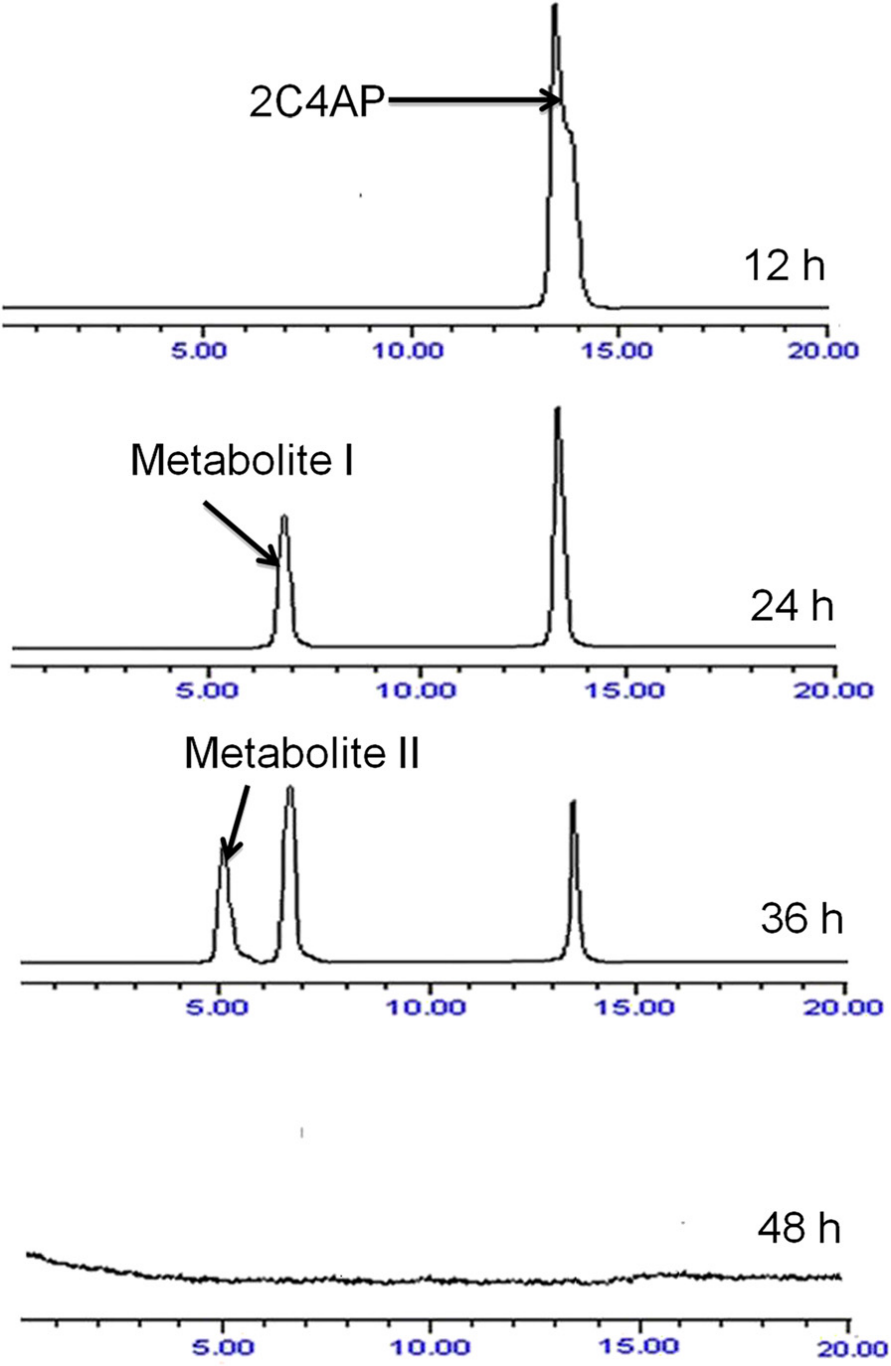
**High performance liquid chromatography eluction profiles of the samples of the degradation of 2-chloro-4-aminophnol by** ***Arthrobacter*** **sp. SPG.**

High performance liquid chromatography analysis of the samples collected at different intervals showed complete disappearance of the peak of 2C4AP within 48 h (Figure 2). In the samples of 0 and 12 h, a single peak of 2C4AP was detected with the retention time of 13.36 min. However, in the sample of 24 h, a peak with retention time 6.89 min (referred to as metabolite 1) appeared along with the peak of the 2C4AP. In the sample of 36 h, the peak of metabolite 11 with the retention time of 5.09 min was detected along with the peaks of metabolite 1 and the 2C4AP. In the sample of 48 h, the peaks of both metabolites and the 2C4AP have disappeared suggesting the complete degradation of 2C4AP by *Arthrobacter* sp. SPG. The retention time of metabolite I and II were matched with authentic chlorohydroquinone (CHQ) and hydroquinone (HQ), respectively.

To identify both metabolites, samples (24 and 36 h) were analyzed with gas chromatography–mass spectrometry (GC-MS). The results of GC-MS analysis showed that the molecular ions of the metabolite I and II were observed at *m/z* 144 and *m/z* 110, respectively. The mass fragment patterns of metabolite I and II were equivalent with that of authentic standards of CHQ and HQ. On the basis of analytical analysis of the samples by HPLC and GC-MS, metabolite I and II were identified as CHQ and HQ, respectively.

### Enzyme assays

We have detected enzyme activities of a 2C4AP-deaminase, a CHQ-dehalogenase and a HQ-dioxygenase in the crude extracts of 2C4AP induced cells of strain SPG. The 2C4AP-deaminase activity showed the stoichiometric release of ammonium ions and the resulting product was identified as CHQ by the GC-MS. The CHQ-dehalogenase activity showed the stoichiometric release of chloride ions and the resulting product was detected as HQ by GC-MS. The HQ dioxygenase activity showed the disappearance of the peak of HQ at 289 nm with appearance of the peak of *γ*-hydroxymuconic semialdehyde, a ring-cleavage product of HQ at 320 nm suggesting the ring-cleavage of the HQ into *γ*-hydroxymuconic semialdehyde.

### Microcosm studies

**Figure 3:**
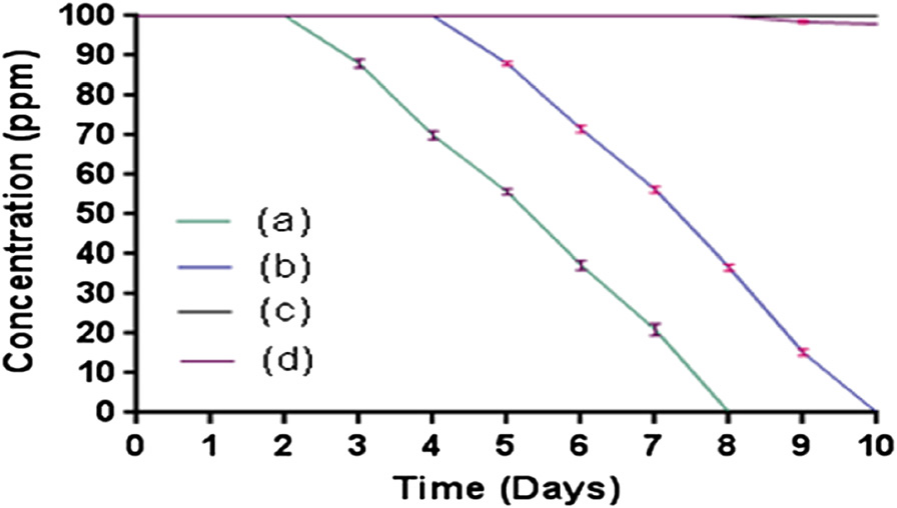
**Degradation of 2C4AP by** ***Arthrobacter*** **sp. SPG in (a) microcosm with sterile soil, (b) microcosm with non-sterile soil, (c) Un-inoculated control with sterile soil and (d) Un-inoculated control with non-sterile soil.**

Microcosm studies showed that the cells of strain SPG were able to degrade 2C4AP in microcosms with sterile as well as non-sterile soil within 8 and 10 days, respectively (Figure 3). No degradation was found at initial 2 days in sterile soil whereas in test microcosm with non-sterile soil, no degradation was observed at initial 4 days. In the sterile soil, the degradation was 12% at 3 days and 30% at 4 days. After 6 days, the degradation rate was 65% in sterile soil whereas almost complete degradation was observed at 8 days in test microcosm with sterile soil. In the test microcosm with non-sterile soil, the degradation was 30%, 45%, 65%, and 85% in 6 days, 7 days, 8 days and 9 days, respectively. The complete degradation of 2C4AP was observed at 10 days in test microcosm with sterile soil. In both of the control microcosms, with sterile and non-sterile soil, there was no degradation within 10 days. GC-MS analysis of the samples showed the presence of CHQ and HQ in the 6 days sample of sterile soil microcosm and the 7 days sample of the non-sterile soil microcosm. No intermediate was detected in any other sample. All microcosm experiments were performed under optimum conditions which include pH 7.0, temperature 30°C, 100 ppm 2C4AP and the inoculum bacterial size 2 × 10^8^ CFU g^−1^ soil.

## Discussion

*Arthrobacter* sp. SPG utilized 2C4AP as its sole source of carbon and energy and degraded it with release of stoichiometric amounts of chloride and ammonium ions. The CHQ was detected as the initial metabolite of the degradation pathway of 2C4AP. The enzyme 2C4AP-deaminase was responsible for the CHQ formation in the 2C4AP degradation. Literature studies showed that the CHQ is a common metabolite in the degradation pathway of several chlorinated compounds [9-13]. Reddy *et al*. [11] reported that the CHQ was degraded either via HQ or via 2-chlorotrihydroxybenzene in degradation pathway of 2,4,6-trichlorophenol in *Phanerochaete chrysosporium*. Miyauchi *et al*. [12,13] showed that CHQ cleaved to maleylacetate or dechlorinated to HQ in the degradation pathway of gamma-hexachlorocyclohexane in *Sphingomonas paucimibilis* UT260. *Arthrobacter* sp. SJCon degraded 2C4NP via a CHQ pathway in which CHQ was cleaved to maleylacetate by a CHQ-dioxygenase [14]. *Burkholderia* sp. RKJ 800 [9] and *Rhodococcus imtechensis* RKJ300 [10] degraded 2C4NP via a CHQ pathway in which CHQ dehalogenated to HQ. In this study, we have also detected HQ as a metabolite of degradation of 2C4AP by strain SPG. Furthermore, we have also detected the activity of the CHQ dehalogenase in the crude extract of the 2C4AP-induced cells of strain SPG that confirmed the formation of the HQ from CHQ with release of chloride ion. The HQ was also detected as a metabolite of degradation pathway of 4-nitrophenol in a various Gram-positive bacteria [7,14,15]. In the degradation pathway of 4-nitrophenol, HQ was cleaved into gamma-hydroxymuconic semialdehyde by a HQ-dioxygenase [7,14]. We have also detected the HQ-dioxygenase activity in the crude extracts of the 2C4AP-induced cells of strain SPG that suggested the cleavage of HQ into gamma-hydroxymuconic semialdehyde.

**Figure 4:**
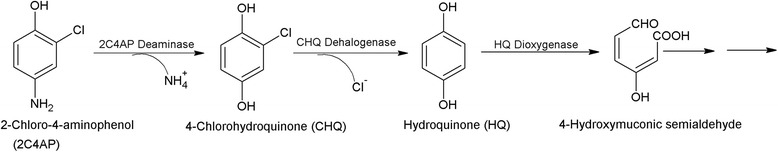
**Proposed pathway of degradation of 2-chloro-4-aminophenol for** ***Arthrobacter*** **sp. SPG.**

On the basis of the identified metabolites and the results of the enzyme assays, we have proposed the 2C4AP degradation pathway for *Arthrobacter* sp. SPG (Figure 4). The 2C4AP degradation was initiated with release of ammonium ion and the formation of CHQ that further dehalgenated to HQ with release of chloride ion. The further degradation of HQ was proceeded via ring cleavage.

The 2C4AP degradation pathway identified in *Arthrobacter* sp. SPG was compared with the degradation pathway of 4C2AP in *Burkholderia* sp. RKJ 800 [1]. It was observed that the initial mechanism of degradation of 2C4AP in strain SPG was similar with that of the 4C2AP degradation in strain RKJ 800 [1]. Both strains initiated the degradation of an isomer of CAPs with removal of ammonium ion by a deaminase [1]. The difference in the degradation pathways of 2C4AP and 4C2AP is that the chloride release was occurred before the ring cleavage in the degradation pathway of 2C4AP whereas in the degradation of 4C2AP, chloride was released after the ring cleavage [1].

The cells of strain SPG were capable of degrading 2C4AP in the soil under laboratory conditions. The rate of the 2C4AP degradation was faster in sterile soil than non-sterile soil. The 2C4AP degradation pattern was very similar in sterile and non-sterile soil. The major difference is an extended lag phase of strain SPG in non-sterile soil due to the presence of natural microbiota. These data indicate that strain SPG initially compete with natural microbiota in non-sterile soil, then acclimated and degraded 2C4AP. Literature studies showed that two kinds of mechanisms may involve in microcosm degradation studies with non-sterile soils; (i) bacterial inhibition in which indigenous microorganisms produced antagonistic substances to inhibit the growth of an inoculated bacterium [14,16] and (ii) microbial cooperation in which indigenous microorganisms enhanced the degradation rate by utilizing xenobiotics or their metabolites [14,17]. In our study, we have not observed the enhancement of degradation rate in non-sterile soil that indicated there is no role of indigenous microbiota to enhance the 2C4AP degradation.

Previous studies showed that several bacteria have ability to degrade various chlorophenols in microcosms with sterile and non-sterile soil [1,4,9,18,19]. *Rhodococcus imtechensis* RKJ300 degraded 2C4NP in soil and the degradation rate was faster in the non-sterile soil than sterile soil [14,18]. Similar findings have been observed in a 4C2NP-mineralizing bacterium, *Exiguobacterium* sp. PMA that degraded 4C2NP with faster rate in non-sterile soil [4]. The equal rate of the degradation of 4C3NP in sterile and non-sterile soil was observed in *Pseudomonas* sp. JHN [19]. Another bacterium, *Burkholderia* sp. RKJ 800 degraded 4C2AP and 2C4NP in soil and the degradation rate of the both compounds was faster in non-sterile soil as compared to sterile soil [1,9]. In this study, strain SPG degraded 2C4AP more rapidly in sterile soil than non-sterile soil.

## Conclusions

*Arthrobacter* sp. SPG utilized 2C4AP as its sole source of carbon and energy and degraded it via a novel pathway with the formation of CHQ and HQ as the metabolites. Initially, a 2C4AP deaminase catalyzed the novel conversion of 2C4AP to CHQ with release of the ammonium ion. In the next step, a CHQ-dehalogenase converted CHQ to HQ with release of chloride ion. The further degradation of HQ occurred via the ring-cleavage. The characteristics feature of this pathway is that both ammonium and chloride ions were released before the ring-cleavage. The potential of strain SPG to degrade 2C4AP in the soil was also investigated and observed that cells of strain SPG degraded 2C4AP efficiently in sterile as well as non-sterile soil. This strain is a good candidate for bioremediation of 2C4AP-contaminated sites. This is the first report of the 2C4AP degradation by any bacteria.

## Material and methods

### Chemicals

2C4AP, HQ and CHQ were purchased from Sigma-Aldrich. All other chemicals used were of high quality.

### Growth and degradation studies

The overnight grown cells of strain SPG (1%, v/v) were inoculated into 500 ml Erlenmeyer flask containing 200 ml minimal medium and 0.3 mM 2C4AP as the sole source of carbon and energy. Samples collected at regular intervals (0 h, 4 h, 8 h, 12 h, 16 h, 20 h, 24 h, 28 h, 32 h, 36 h, 40 h, 44 h, 48 h, 52 h and 56 h) were used for measuring the growth of cells of strain SPG, the 2C4AP degradation and analysis of chloride and ammonium ions. The growth of the cells of strain SPG was directly calculated by spectrophotometer at the absorbance of 600 nm. For degradation studies, samples were centrifuged and extracted with ethyl acetate and the extracted samples were dissolved in the 20 μl methanol and analyzed by high performance liquid chromatography using previously described method [1].

For the analysis of the chloride and ammonium ions, samples collected at regular intervals were centrifuged and supernatants were used to detect the chloride and ammonium ions. Ammonium ions were analyzed using Sigma Ammonia Assay kit. This kit is based on the reaction of the ammonia with *α*-ketoglutaric acid in the presence of L-glutamate dehydrogenase to form L-glutamate. During this reaction, NADPH is oxidized to NADP^+^ [20]. The ammonia concentration was measured by the decrease in absorbance at 340 nm because of the oxidation of NADPH [20]. The chloride ions were analyzed using QuantiChrom™ Chloride assay kit (DICL-250; BioAssay Systems, Hayward, CA, USA) [21]. The chloride ions present in the sample react with the mercuric 2,4,6-tripyridyl-s-triazine to form a colored complex. The color intensity of this compound was measured at 610 nm that is directly proportional to the chloride concentration in the sample [21].

### Identification of metabolites

The overnight-grown cells of strain SPG (1%, v/v) was inoculated into 1 L Erlenmeyer flask containing 500 ml minimal medium and 0.3 mM 2C4AP as the sole source of carbon and energy. Samples (50 ml) collected at regular intervals (0 h, 12 h, 24 h, 36 h, 48 h) were centrifuged and extracted with ethyl acetate. For extraction, the supernatant was mixed with equal volume of ethyl acetate in a separating funnel and shaked for 10–15 minutes [1]. After shaking, the ethyl acetate layer and aqueous layer were allowed to separate [1]. The ethyl acetate layer was collected and referred to as neutral extract [1]. The pH of the aqueous phase was then adjusted to 2.0 with 5 N HCl and was re-extracted with equal volume of ethyl acetate [1]. After separation of two layers, the ethyl acetate layer was collected which was referred to as acidic extract and the aqueous layer was discarded [1]. The neutral and acidic extracts were mixed together and evaporated to dryness using an evaporator at 45°C [1]. The authentic standards (CHQ and HQ) were also extracted. For this, CHQ (0.3 mM) or HQ (0.3 mM) was added into 250 ml Erlenmeyer flask containing 50 ml minimal medium and sample was extracted as described above. The extracted samples were dissolved in 50 μl methanol and analyzed using high performance liquid chromatography (HPLC) and gas chromatography–mass spectrometry (GC-MS).

HPLC analysis was performed using a Waters 600 model high performance liquid chromatography (HPLC) equipped with a photodiode array UV detector set at 300 nm [1]. The stationary phase was a C_18_ column and the injection volume was 15 μl. The mobile phase flow rate was 1.0 mL/min and the mobile phase was composed of methanol (1% glacial acetic acid) and water (1% glacial acetic acid) at a ratio of 80:20.

GC-MS analysis was carried out using a GC-MS-QP5000 instrument (Shimadzu, Tokyo, Japan) equipped with quadrupole mass filter [9]. The separation was performed using DB-1 capillary column with ionization of 70 eV. The column temperature was initially increased from 50°C to 190°C at the rate of 15°C/min and then from 190°C to 280°C at the rate of 10°C/min. The carrier gas (nitrogen) flow rate was 15 ml/min.

### Preparation of crude extracts and enzyme assays

The cells of strain SPG were grown in 1 L Erlenmeyer flask containing 500 ml minimal medium, 0.3 mM 2C4AP and 20 mM sodium succinate. The 24 h-grown cells of strain SPG were centrifuged and the pellet was washed with phosphate buffer (50 mM, pH 7.5) and re-suspended in the same buffer. The cells were sonicated and the cell extracts were centrifuged at 4°C for 15 min to remove cell debris [9]. The resulting supernatant was used for enzyme assays. The 2C4AP-deaminase activity was determined by measuring ammonia released from 2C4AP upon incubation with crude extract. The standard reaction mixture contained 50 mM phosphate buffer (pH 7.5), 0.2 mM NADH, 30 mg protein (crude extract) and 300 μM 2C4AP in a total reaction volume of 2 ml. The reaction mixture was centrifuged upon incubation of 5 min and subjected for ammonia release and the GC-MS analysis as described above. The activities for CHQ-dehalogenase and the HQ dioxygenase were determined by the methods as described previously [9].

### Microcosm studies

The experiments for microcosm studies were performed as described previously [1,4,9]. The composition of the soil used in this study was also similar as described previously [1]. The optimum conditions for degradation of 2C4AP were determined using the strain SPG and the chemical 2C4AP as described previously [1,4,9,18]. For microcosm studies, following four experiments with soil microcosms spiked with 100 ppm 2C4AP were performed (a) test microcosm with sterile soil in which cells of strain SPG were inoculated in the autoclaved soil, (b) test microcosm with non-sterile soil in which cells of strain SPG were inoculated in natural soil, (c) control microcosm with sterile soil where no bacterial strain is inoculated in the autoclaved soil, and (b) control microcosm with non-sterile soil where no bacterial strain is inoculated in natural soil. All soil microcosms were prepared in the 100 ml beaker containing 20 g soil and covered with perforated aluminum foil. These microcosms were inoculated at 30°C for 10 days. The analysis of the 2C4AP degradation in soil was performed as described previously [1,9,18,19]. Samples were also analyzed by the GC-MS to identify the metabolites formed in the soil due to degradation of 2C4AP by strain SPG.
